# Geomicrobiological linkages between short-chain alkane consumption and sulfate reduction rates in seep sediments

**DOI:** 10.3389/fmicb.2013.00386

**Published:** 2013-12-12

**Authors:** Arpita Bose, Daniel R. Rogers, Melissa M. Adams, Samantha B. Joye, Peter R. Girguis

**Affiliations:** ^1^Department of Organismic and Evolutionary Biology, Harvard UniversityCambridge, MA, USA; ^2^Department of Marine Sciences, University of GeorgiaAthens, GA, USA

**Keywords:** short-chain alkanes, methane, ethane, propane, butane, Gulf of Mexico, microbial sulfate reduction, C_1_−C_4_ hydrocarbons

## Abstract

Marine hydrocarbon seeps are ecosystems that are rich in methane, and, in some cases, short-chain (C_2_–C_5_) and longer alkanes. C_2_–C_4_ alkanes such as ethane, propane, and butane can be significant components of seeping fluids. Some sulfate-reducing microbes oxidize short-chain alkanes anaerobically, and may play an important role in both the competition for sulfate and the local carbon budget. To better understand the anaerobic oxidation of short-chain *n*-alkanes coupled with sulfate-reduction, hydrocarbon-rich sediments from the Gulf of Mexico (GoM) were amended with artificial, sulfate-replete seawater and one of four *n*-alkanes (C_1_–C_4_) then incubated under strict anaerobic conditions. Measured rates of alkane oxidation and sulfate reduction closely follow stoichiometric predictions that assume the complete oxidation of alkanes to CO_2_ (though other sinks for alkane carbon likely exist). Changes in the δ^13^C of all the alkanes in the reactors show enrichment over the course of the incubation, with the C_3_ and C_4_ incubations showing the greatest enrichment (4.4 and 4.5‰, respectively). The concurrent depletion in the δ^13^C of dissolved inorganic carbon (DIC) implies a transfer of carbon from the alkane to the DIC pool (−3.5 and −6.7‰ for C_3_ and C_4_ incubations, respectively). Microbial community analyses reveal that certain members of the class Deltaproteobacteria are selectively enriched as the incubations degrade C_1_–C_4_ alkanes. Phylogenetic analyses indicate that distinct phylotypes are enriched in the ethane reactors, while phylotypes in the propane and butane reactors align with previously identified C_3_–C_4_ alkane-oxidizing sulfate-reducers. These data further constrain the potential influence of alkane oxidation on sulfate reduction rates (SRRs) in cold hydrocarbon-rich sediments, provide insight into their contribution to local carbon cycling, and illustrate the extent to which short-chain alkanes can serve as electron donors and govern microbial community composition and density.

## Introduction

Marine hydrocarbon seeps are natural features that support considerable biological diversity and activity (Muyzer and Van Der Kraan, 2008 and references therein). Tectonic activity forms faults that facilitate the release of methane (CH_4_) and other hydrocarbons from deep subsurface oil and gas deposits, as well as gas hydrates (Muyzer and Van Der Kraan, 2008). Methane is a key constituent of the carbon cycle as it is one of the final products of the microbial degradation of organic matter (Thauer et al., 2008 and references therein), though it can also be produced abiotically through thermochemical and geogenic reactions (Horita and Berndt, 1999).

Because CH_4_ is a potent greenhouse gas, there is considerable interest in determining the fate of both biogenic and abiotic methane (for review see Reeburgh, 2007; Thauer et al., 2008). Consequently, microbial methane oxidation under both aerobic and anaerobic conditions has received considerable attention (Thauer et al., 2008 and references therein). The anaerobic oxidation of methane, or AOM, has been the subject of research for four decades, and much of the work has been focused on identifying the responsible microbes, their distribution, and the biochemistry of AOM. To date, five distinct mechanisms of AOM have been discovered (Callaghan, 2013 and references therein; Haroon et al., 2013). The AOM mechanism most relevant to this study is mediated by microbial consortia of archaea, related to the archaeal anaerobic methane oxidizer group ANME, and bacterial sulfate-reducers of the Desulfosarcinales/*Desulfococcus* (DSS) group (Knittel and Boetius, 2009 and references therein). This microbial consortium mediates coupled AOM and sulfate reduction, though the exact nature of the association is not fully understood (Knittel and Boetius, 2009; Milucka et al., 2012 and references therein).

The study of C_2_–C_5_ hydrocarbon degradation has lagged behind that of CH_4_ in spite of their abundance in some environments. Analogous to methane, C_2_–C_5_ gases are formed due to thermal cracking of fossilized organic deposits, and C_1_–C_2_ gases are also produced biologically (Lorant and Behar, 2002; Hinrichs et al., 2006; Jones et al., 2008; Xie et al., 2013). At some sites, including the Gulf of Cadiz, the Gulf of Mexico (GoM), the Caspian Sea, the Monterey Bay canyon (Lorenson et al., 2002), and the Norwegian continental shelf (Hovland and Thomsen, 1997), C_2_–C_5_ hydrocarbons can account for 14–38% of the total gas (see Milkov, 2005 and references therein). In these areas, the oxidation of C_2_–C_5_ hydrocarbons may be a significant contributor to the community bioenergetics of marine seeps (Lorenson et al., 2002; Formolo et al., 2004; Sassen et al., 2004; Alain et al., 2006). For example, at GoM cold seeps, sulfate reduction rates (SRRs) are higher than can be accounted for by AOM alone, indicating that sulfate reduction is linked to the oxidation of other organic compounds potentially including short-chain alkanes (Joye et al., 2004; Orcutt et al., 2010; Bowles et al., 2011). The extent of this as well as the influence of short-chain hydrocarbon oxidation on AOM is poorly constrained, but it is possible that C_2_–C_5_ hydrocarbon degradation is a significant process that co-occurs with AOM, and may compete for a common oxidant (i.e., SO^2−^_4_) (Joye et al., 2004; Orcutt et al., 2010; Bowles et al., 2011).

Recent studies of marine and terrestrial seeps, as well as marine hydrothermal vents, have observed the microbial oxidation of short-chain alkanes coupled to sulfate reduction across a range of temperatures. The microbes, as revealed by phylogenetic analyses of isolates as well as enrichments, align with sulfate reducers within the Deltaproteobacteria and the Firmicutes (Kniemeyer et al., 2007; Savage et al., 2010; Adams et al., 2013; Jaekel et al., 2013). Kniemeyer et al. isolated a bacterium, BuS5, allied to the DSS group within the Deltaproteobacteria that can anaerobically oxidize propane and *n*-butane while reducing sulfate (Kniemeyer et al., 2007). Savage et al. showed that propane and *n*-pentane degrading enrichments from a terrestrial hydrocarbon seep were also dominated by the DSS group (Savage et al., 2010). Jaekel et al. further characterized propane and butane degrading sediment-free enrichments to expand the understanding of the physiology of these microbes (Jaekel et al., 2013). Unlike these previous studies, Adams et al. observed appreciable rates of ethane degradation coupled to sulfate reduction in *ex situ* sediment slurry-based batch reactors with sediments from the Middle Valley hydrothermal vent field (Adams et al., 2013). Collectively, these studies have placed some constraints on the relationship between alkane oxidation and sulfate reduction, though little remains known about the stoichiometric relationship between alkane oxidation—including methane- and sulfate reduction by mixed, natural communities, and their impact on local carbon cycling.

The northern slope of the GoM is an ideal site to study the anaerobic consumption of short-chain alkanes because the sediments lie over hydrocarbon deposits including structure II and H gas hydrates rich in C_1_–C_5_ gases (Joye et al., 2004). The sites of hydrocarbon seepage in the GoM are also characterized by the presence of mats dominated by the chemoautotrophic sulfur oxidizing bacterial genus *Beggiatoa* (Joye et al., 2004). The presence of these microbes suggests that H_2_S is available in the environment, which may indicate high advection and seepage rates (Joye et al., 2004). At the northern slope of the GoM, methane is the dominant component of seeping gas (72–96%) with some contribution from short-chain alkanes. The abundance of the short-chain alkanes decreases with chain length with ethane comprising 2.4–12.4% of the total gas, followed by propane and butane (iC_4_ + nC_4_) (1.2–12.6 and 0.3–4.3%, respectively) (Sassen et al., 1998). Stable carbon isotopic properties of the starting materials (vent gas from the deep subsurface), intermediate products (*in situ* gas hydrate and chemosynthetic fauna), and the end products (authigenic carbonates) of their degradation are also known (Sassen et al., 2004). Anaerobic microbial oxidation of C_2_–C_5_ hydrocarbons has been inferred at the site from the enrichment in the δ^13^C of the residual alkane pools (Sassen et al., 2004). In particular, geochemical measurements reveal a preferential degradation of propane, butane, and pentane (Sassen et al., 2004).

To better understand the role of C_2_–C_5_ hydrocarbon degrading organisms in global geochemical cycles, we examined microbially mediated alkane consumption and SRRs, and the effect of alkane consumption on the inorganic carbon pool using sediments collected from a marine hydrocarbon-rich seep in the GoM. Specifically, we conducted a series of experiments in *ex situ* batch reactors to examine: (1) the rate at which microbial communities degrade C_1_–C_4_ alkanes; (2) the relationship between alkane degradation and sulfate reduction; (3) the degree to which microbially mediated alkane degradation influences the isotopic signatures of the alkanes and dissolved inorganic carbon (DIC) pools; and (4) how community composition of the GoM sediments are affected by the addition of C_1_–C_4_ alkanes. This study advances our understanding by quantifying the potential rates of C_1_–C_4_ alkane consumption, sulfate reduction and the possible effects on the local carbon pool at a well-studied marine habitat. Furthermore, we describe the microbial phylotypes that are most abundant during active C_1_–C_4_ degradation.

## Materials and methods

### Study site and sample collection

Sediments were collected from the Garden Banks mud volcano site (GB425) in the northern GoM (27–33.140 N, 92–32.437 W) at 597 m depth, during an expedition with the *R/V Atlantis* and *DSV Alvin* (Dive 4645) in November 2010. Intact sediment cores were recovered with polyvinylchloride core sleeves (20–30 cm height, 6.35 cm ID, 0.32 cm sleeve thickness). Sediment sampling sites were selected based on the presence of chemoautotrophic *Beggiatoa* mats overlying the sediments and the previous detection of alkanes. Retrieved cores were sealed under Argon gas to limit gas exchange with the atmosphere and to prevent reoxidation of sulfide to sulfate, and refrigerated for transport to the laboratory. It is important to note that sulfide reoxidation is very rapid in these sediments, particularly in sediments hosting microbial mats (Bowles et al., 2011). Therefore, it is not surprising that there is no observable sulfate gradient. Hydrogen concentrations at the study site were determined using a “reduction gas analyzer” as described by Orcutt et al. (2005). The *in situ* SRRs were measured shipboard as described previously (Bowles et al., 2011). The bottom water temperature at this study site was 8°C.

### Batch reactor set-up and sampling

**Figure 1:**
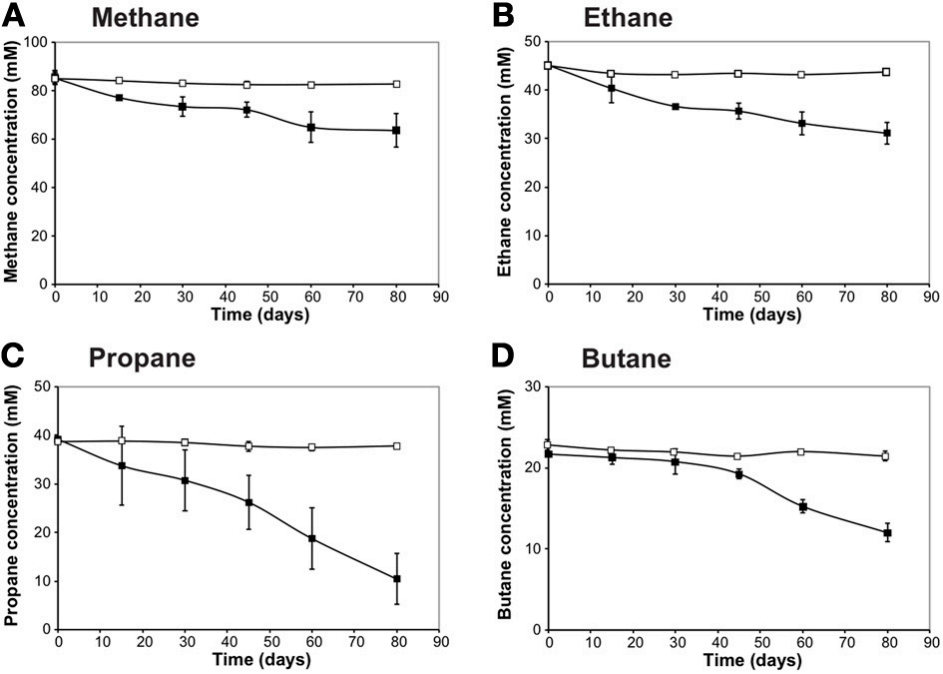
**Alkane consumption as a function of time by the Gulf of Mexico (GoM) site GB425 (dive 4645 and core 22) incubations**. The alkane concentration in the headspace of the sediment incubations was measured using a Hewlett Packard 5890 Series II gas chromatograph equipped with a flame ionization detector, on a Restek Rt-XL Sulfur packed column. **(A)** methane; **(B)** ethane; **(C)** propane; and **(D)** butane. Rates of alkane consumption were calculated using all available time points based on a linear regression.

Collected sediments were transferred to an anaerobic chamber with a 5% H_2_/75% N_2_/20% CO_2_ atmosphere (Coy Laboratory Products, Grass Lake, MI). The sediment core used for this study was stored for 3 months at 7°C. Sediments were thoroughly mixed, and were then diluted with an equal volume of anaerobic medium with 28 mM sodium sulfate and 2 mM sodium sulfide (Widdel and Bak, 1992). No nitrate or nitrite was added to the medium. The resulting slurry was aliquoted into sterile 200 mL serum bottles and sealed with a butyl rubber stopper under strict anoxic conditions. The serum bottle headspace (100 mL) was flushed and then filled with a single gas at ~69 kPa (concentrations of 40–80 mM, Figure 1) of chemically pure (>99% purity) methane, ethane, propane, or butane (Airgas East, Waterford, CT, USA). The solubility at standard conditions (in water) of each gas is as follows: methane (0.9 mM), ethane (1.3 mM), propane (1 mM), and butane (0.8 mM) (webbook.nist.gov/) and exceeds the observed *in situ* concentrations of ~400, 20, 1, and 1 μ M, respectively reported below. Excess gas pressure was used to overcome potential issues in isotopic data interpretation as reported for anaerobic propane oxidation previously (Quistad and Valentine, 2011), and to avoid substrate limitation. The control bottles were flushed and filled with chemically pure (>99% purity) nitrogen (N_2_) gas. An initial sample of each gas (except N_2_) was taken for isotopic analyses. The slurry was also sub-sampled and frozen at −80°C for DNA extraction, sulfide and sulfate quantification.

To test whether alkanes could be oxidized without concomitant sulfate reduction, we use the competitive inhibitor molybdate to inhibit sulfate reduction and monitored the subsequent alkane consumption rates. Briefly, 5 mL of sediment slurry was transferred to 25 mL Balch tubes, in duplicate, per gas amendment, and sealed using a rubber butyl stopper inside an anaerobic chamber. These incubation volumes were used to maximize analyses given the limited sediment volumes. Due to the high sulfate concentration present in these incubations, the affect of changing the incubation volume should not be detrimental to sulfate reduction. Sodium molybdate was added to each tube to a final concentration of 28 mM (Orcutt et al., 2008). These tubes were then flushed and filled with the appropriate C_1_–C_4_ or N_2_ gas at ~69 kPa.

All the reactors were incubated at 7°C and the headspace was sampled every 15-days to monitor C_1_–C_4_ consumption. After 80 days of incubation, the final gas concentrations were measured, and gas samples were archived in gastight Exetainers (Labco International, Houston, TX, USA) for natural abundance isotopic measurements. Samples were also withdrawn for DIC, DNA, sulfide and sulfate measurement, and preserved by freezing at −80°C in appropriate vials. All sub-samples were collected and measured in triplicate.

### Geochemical measurements

C_1_–C_4_ alkanes were quantified from the headspace by subsampling a 50 μL aliquot and analyzing alkane concentrations on a gas chromatograph (Hewlett Packard 5890 Series II) equipped with a flame ionization detector and a packed column (RestekRt-XL). Chemically pure alkanes (>99% purity) (Airgas East, Waterford, CT, USA) were used to generate standard curves. To account for potential alkane leakage from the bottles, we set up sediment-free controls and monitored changes in alkane concentration over time. Sediment-free controls showed ~4 ± 1% variation in gas measurements, which represents both the analytical resolution of our measurements and/or modest loss of gas due to leakage or sorption into the stoppers, and is well below the rates of loss observed in the biological treatments (see below). Sulfate concentrations were determined using the QuantiChrom™ Sulfate Assay Kit (BioAssay Systems, Hayward, CA, USA). Sulfide concentrations were measured using a colorimetric assay based on the Cline method (Cline, 1969). Nitrate was measured using the resorcinol method as described previously (Zhang and Fischer, 2006). Nitrite was measured as previously described (Pai et al., 1990).

### Microbial sulfate reduction rate measurements

SRRs were measured using a previously described radiotracer method (Fossing and Jorgensen, 1989). Briefly, the slurry incubations were opened under anaerobic conditions and 5 mL subsamples of the enrichments were transferred to a Balch tubes. The tubes were sealed and pressurized as described previously. The Balch tubes were then amended with ca. 10 μ L of Na^35^SO^2−^_4_ (2 μ Ci) and incubated for 1 day. Following incubation the slurries were shaken and 1 mL of slurry was drawn by syringe into 5 mL of 20% zinc acetate and shaken, effectively trapping H^35^_2_S_(aq)_ as ZnS_(s)_. The ZnS solution was placed into a 15 mL Falcon tube, and washed three times with a 3% NaCl solution to remove any residual ^35^SO^2−^_4_. Sulfide was extracted using the hot chromium reduction method (Fossing and Jorgensen, 1989), ultimately trapping sulfide in 20% Zn-acetate. The activity of ^35^S was determined by liquid scintillation and SRR were calculated after Fossing and Jorgensen (1989) using Equation 1.

(1) $$\text{SRR}=\left[{\text{SO}}_{4}^{2-}\right]*\;\frac{1.06}{t}*\frac{a}{\left(A+a\right)}$$

Where [SO^2−^_4_] is the concentration (nmol mL^−1^) of sulfate incubation, *a* is the activity (dpm) of the trapped sulfide, 1.06 is the fractionation factor between the sulfide and sulfate pools, *A* is the activity of the sulfate pool, and *t* is the incubation time (days). The rates are presented in units of nmol S mL^−1^ day^−1^.

### Isotope analysis

All isotopic analyses were performed at the Stable Isotope Facility at University of California, Davis using a method modified from a previous publication (Atekwana and Krishnamurthy, 1998). For DIC measurements, 1 mL filtered (0.2 μm) water samples were collected and injected into evacuated 12 mL septum capped vials (Exetainers, Labco, Houston, TX, USA) containing 1 mL 85% phosphoric acid. The evolved CO_2_ was purged from vials through a double-needle sampler into a helium carrier stream (20 mL min^−1^). For high concentration samples, gases were sampled by a six-port rotary valve (Valco, Houston, TX, USA) with a 100 μL loop programmed to switch at the maximum CO_2_ concentration in the helium carrier. For low concentration samples, the entire CO_2_ content was frozen in a trapping loop then released to the GC column. The CO_2_ was passed to the IRMS through a Poroplot Q GC column (25 m × 0.32 mm ID, 45°C, 2.5 mL/min). A reference CO_2_ peak was used to calculate provisional delta values of the sample CO_2_ peak. Final δ^13^C values were obtained after adjusting the provisional values such that correct δ^13^C values for laboratory standards were obtained. Two laboratory standards were analyzed every 10 samples. The laboratory standards are lithium carbonate dissolved in degassed, deionized water, and a deep seawater reference material (both calibrated against NIST 8545).

For isotopic analyses of the C_1_–C_4_ gases, a ThermoScientific PreCon concentration system interfaced to a ThermoScientific Delta V Plus isotope ratio mass spectrometer (ThermoScientific, Bremen, DE) was used as described previously (Yarnes, 2013). Gas samples were purged from Exetainers through a double-needle sampler into a helium carrier stream (20 mL/min), which is passed through a H_2_O/CO_2_ scrubber [Mg(ClO_4_)_2_, Ascarite] and a cold trap cooled by liquid N_2_. The gas was separated from residual gases by a Rt-Q-BOND GC column (30 m × 0.32 mm × 10 μm, 30°C, 1.5 mL/min). After the gas eluted from the separation column, it was either oxidized to CO_2_ by reaction with nickel oxide at 1000°C (δ^13^C), or pyrolyzed in an empty alumina tube heated to 1350°C (δ^2^H) and subsequently transferred to the IRMS. A pure reference gas (CO_2_ or H_2_) was used to calculate provisional delta values of the sample peak. Final δ-values are obtained after adjusting the provisional values for changes in linearity and instrumental drift such that correct δ-values for laboratory standards were obtained. Laboratory standards were commercially prepared gases diluted in helium or air and were calibrated against NIST 8559, 8560, and 8561.

### DNA extraction, massively parallel sequencing, and phylogenetic analyses

Sediment was subsampled under anoxic conditions for *T*_0_ and *T_f_* for nucleic acid extractions. These samples were flash frozen in liquid N_2_ and stored at −80°C until use. DNA was extracted using the PowerSoil® DNA Isolation Kit (MO BIO Laboratories, Inc., Carlsbad, CA, USA) as per the manufacturer's guidelines. The extracted DNA was subjected to massively parallel sequencing of the 16S ribosomal RNA (rRNA) gene using Roche 454 Titanium™ chemistry and the primer pairs 27F/519R and 340F/806R for the bacterial V1–V3 and archaeal V3–V4 regions, respectively (Dowd et al., 2008; Acosta-Martinez et al., 2010). The resulting sequences were analyzed as previously described, and denoised using the QIIME pipeline (Adams et al., 2013). Phylogenetic analysis was performed as previously described using FastTree(2.1.7) for tree generation with 25 representative sequences (Adams et al., 2013). All sequences generated in this study are deposited with NCBI (accession #SRP032824).

### Quantitative-PCR

Quantitative PCR (qPCR) was used to determine the abundance of bacterial and archaeal 16S rRNA, *dsrA*, *aprA*, and *mcrA* genes. In addition, qPCR was used to enumerate the abundance of sulfate-reducing prokaryotes by amplifying the adenosine 5′-phosphosulfate [APS] reductase (*aprA*) gene with primers specific to sulfate-reducing bacteria and archaea (Christophersen et al., 2011). Primers specific to the bacterial dissimilatory sulfite reductase (*dsrA*) gene were used to quantify members of sulfate-reducing bacteria (Kondo et al., 2004). We refer to all sulfate-reducing microbes as sulfate-reducing prokaryotes or SRP throughout. Methanogenic archaea were quantified using *mcrA* primers directed specifically toward the methanogenic methyl Coenzyme M reductase encoding gene (Luton et al., 2002; Ver Eecke et al., 2012). Standard curves were constructed by serial dilution of linearized plasmids containing the target gene (Table 1). Quantification was performed in triplicate with the Stratagene MX3005p qPCR System (Agilent Technologies) using the Perfecta SYBR FastMix with low ROX (20 μL reactions, Quanta Biosciences, Gaitherburg, MD) and specific primers and annealing temperatures (Table 1). The temperature program for all assays was 94°C for 10 min, 35 cycles of 94°C for 1 min, the annealing temperature for 1 min (Table 1), extension at 72°C for 30 s, and fluorescence read after 10 s at 80°C. Following amplification, dissociation curves were determined across a temperature range of 55–95°C. *C_t_*-values for each well were calculated using the manufacturer's software.

**Table 1:** **Primers and Conditions for quantitative PCR assays**.

**Process**	**Target gene**	**Forward primer (nM)**	**Reverse primer (nM)**	**Positive control**	**Annealing temp in °C (References)**
Sulfate reduction	Dissimilatory sulfite reductase	*DSR1-F+*(400)	*DSR-R* (600)	*Desulfovibrio vulgaris*	58°C (Kondo et al., 2004)
		ATCGGNCARGCNTTYCCNTT	GTGGMRCCGTGCAKRTTGG		
Sulfate reduction	Adenosine 5′-phosphosulfate reductase	*aps3F* (400)	*aps2R* (400)	*Desulfovibrio vulgaris*	55°C (Christophersen et al., 2011)
		TGGCAGATCATGWTYAAYGG	GCGCCGTAACCRTCYTTRAA		
Methanogenesis	Methyl CoM reductase	*qmcrAF-alt (150)* GAR GAC	*ML-R (200)*	*Methanosarcina acetivorans, Methanococcus jannaschii*	59°C (Luton et al., 2002; Ver Eecke et al., 2012)
		CAC TTY GGH GGT TC	TTCATTGCRTAGTTWGGRTAGTT		
Bacteria	16S rRNA	*Bact1369F* (1000) GTT GGG	*Prok1541R* (1000)	*Arcobacter nitrofigulis*	59°C (Suzuki et al., 2001)
		GCC RCC WCK KCK NAC	CGGTGAATATGCCCCTGC		
Archaea	16S rRNA	*Arch349F (500)*	*Arch806R (500*)	*Ferroplasma acidarmonas* Fer1	54°C (Takai and Horikoshi, 2000)
		GYGCASCAGKCGMGAAW	GGACTACVSGGGTATCTAAT		

## Results

### Geochemical characteristics of site GB425

Though the gross geochemistry of this site has been previously described (Joye et al., 2009), here we present the alkane concentrations and other geochemical attributes of the specific sediments used in these studies (Table 2). DIC ranged between 4 and 6 mM, while the observed dissolved organic carbon (DOC) is about 1–3 mM through the sediment depths surveyed. Nitrate and nitrite concentration was 5–40 μ M in the upper layers of the sediment. Sulfate, the dominant oxidant, was replete throughout the sediment profile (24–36 mM) and was higher than typical seawater values (28 mM) (Canfield and Farquhar, 2009). *n*-alkanes were observed only between 9 and 15 cm sediment depth. Between the depth ranges of 9–12 and 12–15 cm ethane was observed at 17.22–22.33 μ M, propane at 1.45–0.75 μ M, butane at 0.74–0.35 μ M. Pentane was not observed. Methane concentrations peaked at 425.05 μ M, at ~15–18 cm sediment depth (Table 3).

**Table 2:** **Geochemical data from site GB425, from which sediments were collected for these analyses in November 2010 (27°33.1887N, 93°32.4449W)**.

**Depth range (cm)**	**DIC (mM)**	**d^13^C-DIC (‰)**	**Hydrogen (nM)**	**DOC (mM)**	**Sulfate (mM)**	**Sulfide (mM)**	**Nitrate and Nitrite (mM)**	**Methane (μM)**	**pH**	**Sulfate reduction rate (nmol mL^–1^ day^–1^)**	**Anaerobic methane oxidation (nmol mL^–1^ day^–1^)**
Overlying water	n.s.	n.s.	n.s.	958	n.s.	n.s.	23.1	n.s.	7.5	n.a.	n.a.
0–3	3.9	−10.0	37.4	2477	24.2	0.6	36.2	122.3	7.5	86 ± 5	0.8 ± 0.3
3–6	4.4	−10.5	242.4	1203	32.3	0.1	5.2	63.0	7.6	344 ± 216	1.6 ± 0.3
6–9	4.2	−12.6	21	1702	32.3	0.6	b.d.l.	378.1	7.9	182	6.8 ± 0.1
9–12	5.1	−19.5	29.8	1.s.	27.9	0.2	b.d.l.	371.9	i.v.	135 ± 22	5.0 ± 2.5
12–15	4.7	−15.2	51.9	1.s.	n.s.	0.8	b.d.l.	413.1	i.v.	n.s.	n.s.
15–18	4.9	−20.1	31.4	1.s.	29.9	0.6	b.d.l.	425.1	8.0	596 ± 8	30.3 ± 7.6
18–21^*^	5.8	−28.6	38.9	2618	29.1	n.s.	40.6	200.2	i.v.	185 ± 216	7.8 ± 0.5
21–24	6.0	−23.7	39.6	2982	25.0	0.1	b.d.l.	357.7	i.v.	743 ± 340	18.2 ± 10.0
24–27	5.3	−23.3	50.7	1420	35.8	1	b.d.l.	288.8	i.v.	287 ± 120	18.9 ± 0.4
27–30	5.3	−24.4	31.5	1.s.	n.s.	1.2	b.d.l.	203.2	i.v.	n.s.	n.s.

*DIC, Dissolved inorganic carbon; DOC, Dissolved organic carbon, n.s., No sample; l.s., Lost sample; i.v., Insufficient volume; b.d.l., Below detection limit; n.a., Not applicable. Rate measurements are mean ± standard error (n = 2)*.

* *Data at this depth range appears unreliable*.

**Table 3:** **C_1_–C_5_ alkane concentrations in sediments at site GB425, from which sediments were collected for this study in November 2010 (27°33.1887N, 93°32.4449W)**.

**Depth range (cm)**	**Methane (μM)**	**Ethane (μM)**	**Propane (μM)**	***n*-butane (μM)**	**Pentane (μM)**
9–12	371.94	17.22	1.45	0.74	0.00
12–15	413.12	22.33	0.75	0.35	0.00

### C_1_−C_4_ alkane oxidation occurs in batch reactors

**Figure 2:**
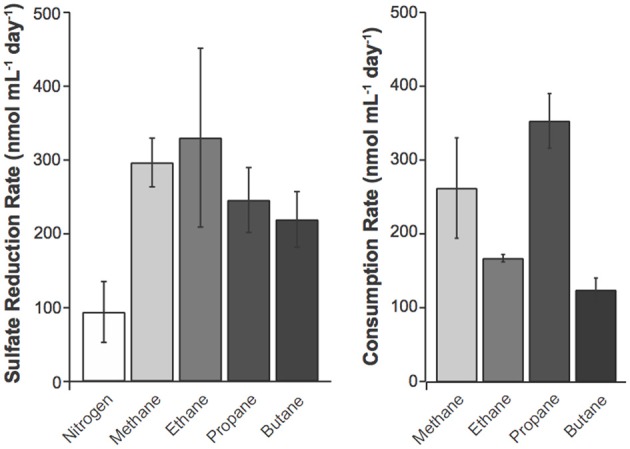
**Potential sulfate reduction rates (SRR) were measured using the ^35^SO^2−^_4_ radiotracer method (Fossing and Jorgensen, 1989) and consumption rates for C_1_–C_4_ alkanes by alkane amended slurries of GoM site GB425 sediments**. The SRR assays were performed for 24 h. Values represent average ± standard deviation of triplicate measurements of duplicate incubations. Alkane consumption rates were calculated from a linear regression as in Figure 1. Rates of sulfate reduction were calculated as described in the methods.

*n*-Alkane consumption began within the first 15 days of the 80-day incubations for C_1_–C_3_ gases (defined as >10% consumption compared to *T*_0_) (Figure 1). C_4_ consumption was only measurable after ~45 days of incubation. The highest % consumption was observed for propane (73 ± 13%) followed by butane (45 ± 5%), ethane (31 ± 6%), and methane (25 ± 6%). The highest rate of consumption was observed for propane (354 ± 37 nmol mL^−1^ day^−1^) followed by methane (263 ± 68 nmol cm^−3^ day^−1^), ethane (168 ± 5 nmol cm^−3^ day^−1^), and butane (125 ± 16 nmol cm^−3^ day^−1^) (Figure 2, Table 4). Along with alkane oxidation we also observed a decline in sulfate concentrations and a concomitant increase in sulfide concentrations (Table 5). Importantly, the sulfide concentrations were below those observed to be inhibitory (16.1 mM) for sulfate-reducing bacteria (Reis et al., 1992).

**Table 4:** **Comparing rate of alkane oxidation and sulfate reduction, and the effect of molybdate on alkane oxidation**.

	**Rate of alkane consumption (nmol mL^−1^ day^−1^)**	**Rate of alkane consumption with molybdate addition (nmol mL^−1^ day^−1^) (% inhibition)**	**Total sulfate reduction rate (nmol mL^−1^ day^−1^)**	**Observed ratio using total SRR alkane:SO^2−^_4_**	**Corrected sulfate reduction rates above the nitrogen control (nmol mL^−1^ day^−1^)**	**Observed ratio using corrected SRR alkane:SO^2−^_4_**	**Predicted ratio of alkane: SO^2−^_4_ from Table 6**
Methane	263 ± 68	29 ± 1 (89)	297 ± 33	0.9	203 ± 53	1.3	1
Ethane	168 ± 5	12 ± 4 (93)	330 ± 121	0.51	236 ± 127	0.7	0.57
Propane	354 ± 37	10 ± 5 (97)	246 ± 44	1.4	152 ± 60	2.3	0.4
Butane	125 ± 16	14 ± 2 (89)	220 ± 38	0.57	125 ± 56	1	0.34

*Rates of alkane consumption were calculated using all available time points based on a linear regression. Rates of sulfate reduction calculated as described in the methods*.

**Table 5:** **Sulfate and sulfide concentrations measured in the initial sediment slurry and at the final time-point**.

	**Sulfate concentration (mM)**	**Sulfide concentration (mM)**
Sediment slurry (Initial)	31.6 ± 1.2	2.3 ± 0.2
N_2_ control (Final)	26.4 ± 1.8	8.2 ± 0.1
Methane (Final)	26.1 ± 6.1	9.1 ± 0.4
Ethane (Final)	22.1 ± 4.0	10.4 ± 1.1
Propane (Final)	15.6 ± 4.1	9.5 ± 1.4
Butane (Final)	13.3 ± 1.2	15.4 ± 3.2

### Sulfate reduction is coupled to C_1_−C_4_ alkane oxidation

The addition of each C_1_–C_4_ gas increased the SRR over the N_2_ control treatment by at least 2-fold (Figure 2). The rates reported from these incubations are comparable to previous reports from GoM non-seep porewaters and sediments (Arvidson et al., 2004; Joye et al., 2004) but lower than those measured shipboard on freshly collected samples (Table 2). While it is impractical to identify the precise cause of this discrepancy, there are a few likely factors that could have contributed to these differences, including (A) natural heterogeneity in the geochemistry and microbial community composition and activity; (B) the process of sediment homogenization prior to incubations, which does not represent maximal or minimal rates; and (C) changes in microbial composition and activity during the 3 months of storage. Comparison of SRR in the one-day incubations to C_1_–C_4_ alkane oxidation rates (Table 4) shows that the addition of methane or any of the four alkanes stimulates SRR over the N_2_ control treatment. From the predicted reaction stoichiometry (Table 6) both the methane and ethane oxidation rates correspond closely with the observed SRR. In contrast substantially higher levels of propane and butane oxidation were observed than can be supported by sulfate reduction alone. Incubation with molybdate inhibited the oxidation of C_1_–C_4_ gases by ~90–97% (Table 4), consistent with the direct involvement of sulfate-reducing prokaryotes in alkane oxidation.

**Table 6:** **Gibbs free energy of the anaerobic oxidation of acetate, methane, and alkanes using sulfate as an electron acceptor (conditions shown are at standard temperature and pressure)**.

	**Sulfate reduction process**	**Reaction**	ΔG^0^ (kJ/mol SO^2−^_4_)^*^	**Carbon source: SSO^2−^_4_**	**C:SO^2−^_4_**
1	Heterotrophic (acetate)	SO^2−^_4_ + CH_3_COO^−^ → 2HCO^−^_3_ + HS^−^	−47.7	1:1	2:1
2	Heterotrophic (methane)	SO^2−^_4_ + CH_4_ → HCO^−^_3_ + HS^−^ + H_2_O	−33	1:1	1:1
3	Heterotrophic (ethane)	14SO^2−^_4_ + 8C_2_H_6_ → 14HS^−^ + 16HCO^−^_3_ + 8H_2_O + 2H^+^	−39.81	8:14	16:14
4	Heterotrophic (propane)	5SO^2−^_4_ + 2C_3_H_8_ → 6HCO^−^_3_ + 5HS^−^ + H^+^ + 2H_2_O	−33.06	2:5	6:5
5	Heterotrophic (butane)	26SO^2−^_4_ + 9C_4_H_10_ + 4H_2_O → 36HCO^−^_3_ + 36H^+^ + 26HS^−^	−14	9:26	18:13
6	Autotrophic (with H_2_)	SO^2−^_4_ + 2HCO^−^_3_ + 8H_2_ + 2H^+^ →CH_3_COO^−^ + HS^−^ + 8H_2_O	−336.5	2:1	2:1

*Autotrophic sulfate reduction, in which hydrogen is used to reduce inorganic carbon, is shown for reference*.

* *Δ G^0^ Values reported are those calculated under standard conditions of 1 M concentrations for soluble reactants, 1 atmosphere pressure for gases, 298.15 K temperature at pH 7.0 and are calculated using values from the CRC Handbook for Chemistry and Physics (http://www.hbcpnetbase.com/)*.

### Carbon isotopic signature and analyses

The δ^13^C signature of the methane in the headspace did not appreciably change over the course of the incubation period (Table S1). This contrasts with the isotopic signatures of the other alkanes. As mentioned, ethane concentration decreased to about 10 mM over the course of the incubation, but the isotopic change in the pool was not significant. The incubations with propane showed the largest decrease in concentration (~30 mM) over the 80-day incubation period. Over this time, the propane pool was enriched by 4.4‰. Finally, incubations with butane resulted in a decrease in the pool size of ~10 mM (1 mmol) and an enrichment in the residual butane pool of 4.5‰.

### Microbial community analyses

#### 454 pyrotag sequencing

**Figure 3:**
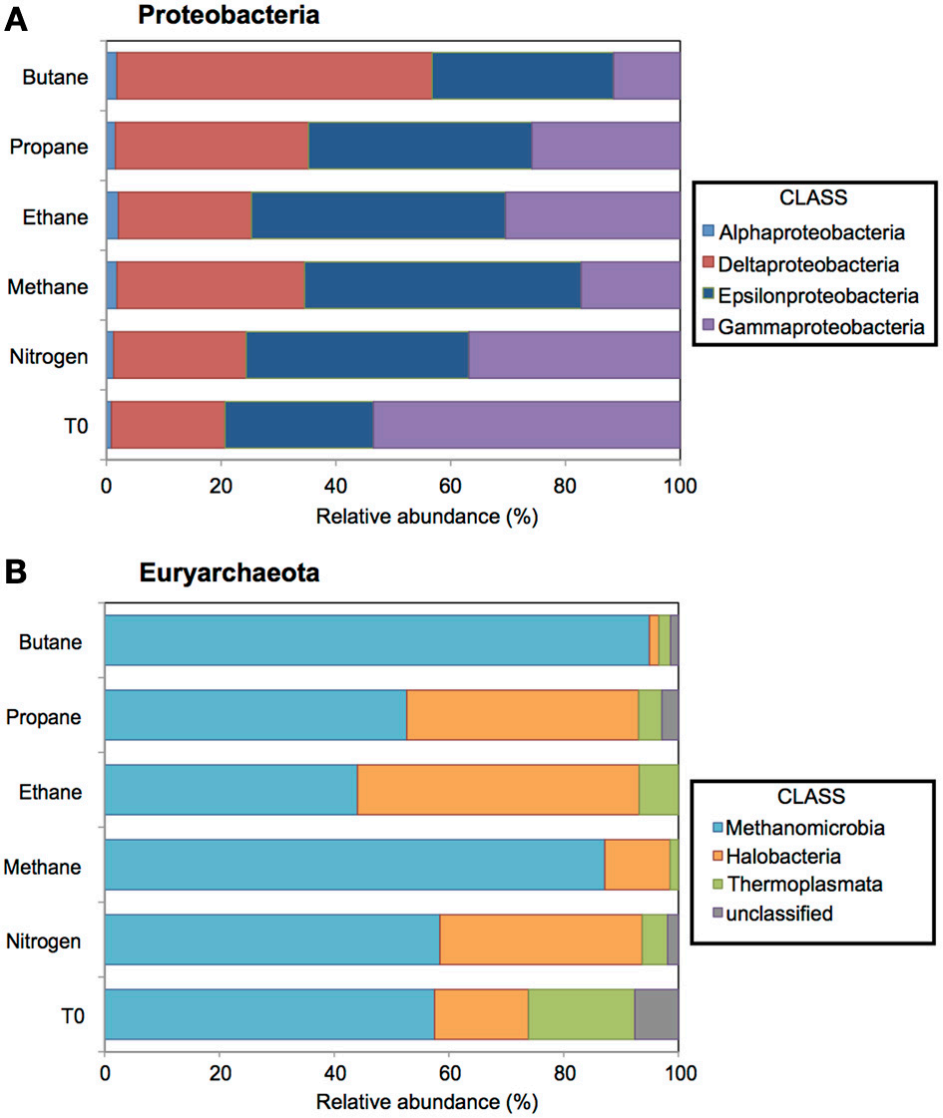
**Relative abundance (percentage) of Proteobacteria and Euryarchaeota determined from massively parallel pyrosequencing of DNA extracted from batch reactor sediments incubated with methane, ethane, propane, butane, and nitrogen and pre-incubation (*T*_0_) sediments**. Top **(A)** and bottom **(B)** panels show the taxonomic breakdown of sequences at the class and order level, respectively. Sequences sharing 97% nucleotide sequence identity are defined as operational taxonomic units (OTUs).

**Figure 4:**
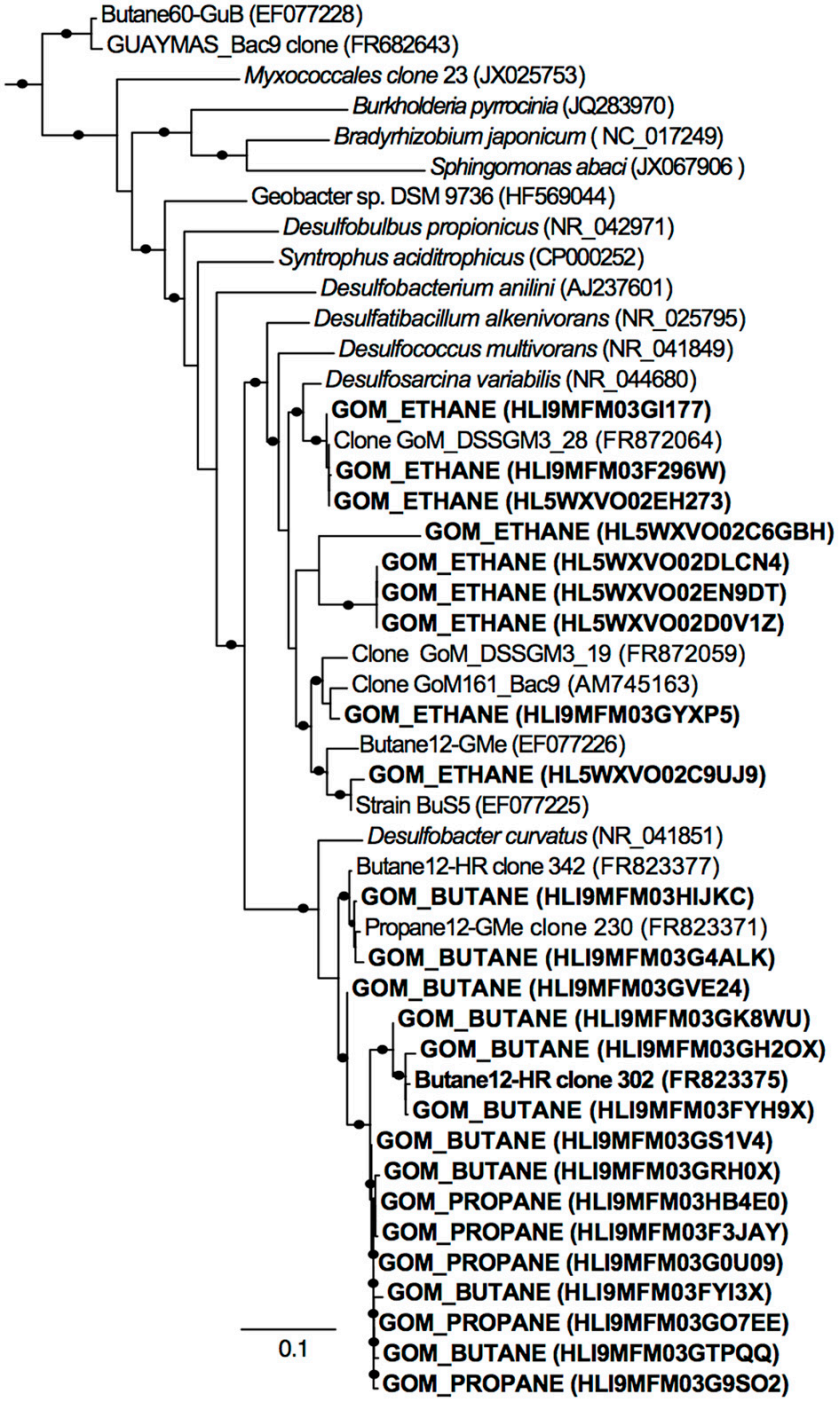
**Phylogenetic affiliation of 16S rRNA Deltaproteobacterial gene sequences retrieved from Gulf of Mexico batch reactor sediments**. A total of 25 representative sequences from Gulf of Mexico sediments incubated in batch reactors with ethane (GOM_ETHANE), propane (GOM_PROPANE), and butane (GOM_BUTANE) are shown in bold. The phylogenetic tree was generated by maximum likelihood with FastTree Version 2.1.3. Local support values shown are based on the Shimodaira–Hasegawa (SH) test with 1000 resamples. Only values >80% are shown on the branches as black circles. The 16S rRNA sequence of *Archaeoglobus profundus* DSM 5631(NR_074522) was used as an outgroup. Scale = 0.1 substitutions per site.

A total of 11,725, 17,003, 12,529, 16,208, and 18,015 bacterial sequences were analyzed from sediments incubated with N_2_, methane, ethane, propane, and butane, respectively, and 12,944 bacterial sequences from the *T*_0_ sediment. There were shifts between the Proteobacterial communities of the alkane batch reactors in comparison to the control and *T*_0_ sediment community (Figure 3A). Among sequences allied to known sulfate-reducing Deltaproteobacteria, there was an increase from the *T*_0_ sequences (~20%) in the N_2_, methane, ethane, propane, and butane sequence libraries (~23, 32, 23, 33, and 55%, respectively) (Figure 3A). In turn, there was a decrease in the representation of Gammaproteobacteria in the N_2_, methane, ethane, propane, and butane sequence libraries (~37, 17, 30, 26, and 12%, respectively) from the *T*_0_ sequences (~53%). 16S rRNA gene phylogeny revealed that the ethane reactors harbored a putative SRP community that was distinct from the propane and butane reactors (Figure 4). These sequences comprised the majority (90–95%) of the Deltaproteobacterial community (Figure 4). In the ethane reactor community, the most closely related Deltaproteobacterial 16S rRNA gene sequences (95–99% nucleotide sequence identity) included strain BuS5 (accession no. EF077225), the enrichment culture “Butane12-GMe” (accession no. EF077226), and other SRP clones from sediments retrieved from the GoM (clone GoM_DSSGM3_28, accession no. FR872064; clone GoM_DSSGM3_19, accession no. FR872059; and clone GoM161_Bac9, accession no. AM745163) (Kniemeyer et al., 2007; Orcutt et al., 2010; Kleindienst et al., 2012). In contrast, SRP sequences in the propane and butane batch reactor communities were most closely allied to uncultured Deltaproteobacteria clones from propane- and butane-oxidizing enrichments of hydrocarbon seep sediments from the GoM (Propane12-GMe clone 230, accession no. FR823371) and Hydrate Ridge (Butane12-HR clone 302, accession no. FR823375 and Butane12-HR clone 342, accession no. FR823377) (Jaekel et al., 2013).

A total of 18,667, 10,291, 18,545, 12,462, 9743, and 13,233 archaeal sequences were also analyzed from the N_2_, methane, ethane, propane, and butane batch reactors and *T*_0_ sediments, respectively. There were notable shifts in the sequences allied to the class Methanomicrobia from the initial sediment community and across the different alkane batch incubations (Figure 3B). Over 58% of sequences were allied to Methanomicrobia in *T*_0_ sediments, increasing to comprise ~87 and 94% of methane and butane sequences. Within the Methanomicrobia, there were also notable changes in sequences identified as phylotypes that mediate AOM. For the putative methane-oxidizing communities, ANME-1 comprised ~40% of the Methanomicrobia in the incubation with methane, but less than 5% of sequences were allied to ANME-1 in the *T*_0_, N_2_, ethane, propane, and butane sediments (Figure S1).

#### Quantitative PCR

**Figure 5:**
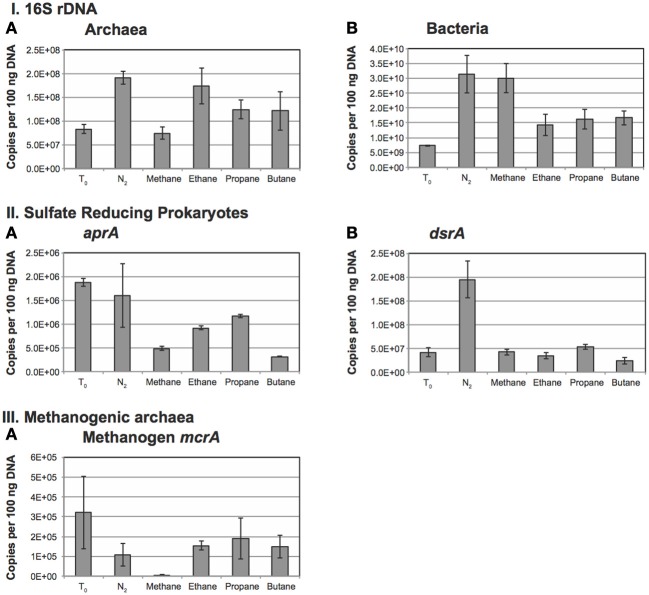
**Abundance of microbes determined using quantitative PCR**. Panel **(I)** represents the 16S rRNA abundances for **A:** Bacteria and **B:** Archaea. Panel **(II)** represents the abundance of sulfate-reducing bacteria as determined using **A:** *aprA* and **B:** *dsrA*. Panel **(III)** represents the abundance of methanogens as determined using **A:** *mcrA*.

qPCR using specific primers for 16S rRNA showed that bacterial 16S rRNA gene abundance was two orders of magnitude higher than archaeal 16S rRNA gene abundance at the start of the incubation (Figures 5IA,B). Bacterial abundance was only slightly elevated (less than an order of magnitude) over the *T*_0_ assessment in all treatments at the end of the incubation period with the greatest increase in population observed in the N_2_ and CH_4_ amendments (~3-fold increase). Addition of alkanes also stimulated bacterial population growth of about 2-fold over the initial population estimates. N_2_ and ethane amendments resulted in a 3-fold increase in archaeal populations while propane and butane yielded a 1.5-fold increase. These differences are consistent among treatments. However, 40% of bacterial genomes contain 1–2 copies of rRNA genes, though microbial genomes with as many as 15 copies have been reported (Acinas et al., 2004). Moreover, archaeal genomes are known to harbor between 1 and 5 rRNA gene copies per genome (Acinas et al., 2004). Thus, given these differences, as well as environmental heterogeneity and other factors, the differences presented here likely reflect relative changes in proportion, but the significance of these changes among treatments remains unconstrained.

Estimates of *aprA* gene abundance, a marker for SRP, reveal the highest abundances at the initiation of the incubation and under the N_2_ amendments. *aprA* gene abundance after incubation with methane or the tested alkanes show a decrease in abundance, perhaps indicating a shift in community due to incubation effects that is consistent with the decrease in bacterial 16S rRNA gene abundance over the same treatments. Notably, of the alkane additions, propane maintained the largest SRP population followed by ethane, methane and butane treatments (Figure 5IIA).

The 16S rRNA gene phylogeny of SRP is diverse and difficult to capture with specific primers. Accordingly, we employed primers targeting the gene encoding for dissimilatory sulfite reductase (subunit A) with primers that target both Gram-positive and Gram-negative bacterial species of SRP (Kondo et al., 2004). With the exception of the N_2_ treatment, the *dsrA* gene abundance is similar across all treatments. The *dsrA* gene abundance in the N_2_ treatment is about 3-fold higher than observed in other treatments (Figure 5IIB).

Archaeal *mcrA* gene abundance was highest, and had the greatest standard deviation at the initial sampling. The lowest observed *mcrA* gene abundance occurred in the methane amended samples and concurs with a similar observed decrease in the total archaeal 16S rRNA gene abundance described above. This suggests that the addition of methane adversely affected the natural methanogen population over the course of the incubation (Figure 5IIIA).

## Discussion

The anaerobic microbial degradation of short-chain alkanes has recently gained attention because microbes mediating these processes may compete for the oxidant pool (sulfate), potentially influencing the rates of AOM (Kniemeyer et al., 2007; Savage et al., 2010; Adams et al., 2013; Jaekel et al., 2013). The data herein reveal that C_1_–C_4_ alkane consumption—including anaerobic ethane oxidation—stimulated sulfate reduction. To assess the significance of the determined potential rates of sulfate reduction and *n*-alkane consumption, two points must be addressed: (1) the intrinsic sulfate reduction activity in the GoM sediments used, and (2) the concentrations of the substrates used relative to those measured *in situ*. First, bulk geochemical analyses show that the GoM sediments are rich in organic matter and hydrogen that can support the growth of heterotrophic and autotrophic SRP. At this study site, the DOC concentration was ~1–3 mM and hydrogen was in the nM range. It was therefore critical to account for sulfate reduction attributable to endogenous electron donors, which we did by maintaining the native sediments under a N_2_ atmosphere. Not surprisingly, these incubations exhibit intrinsic sulfate reduction activity at nearly 100 nmol SO^2−^_4_ mL^−1^ day^−1^. Importantly, the addition of *n*-alkanes increased this baseline sulfate reduction. Second, sulfate concentrations used in the *ex situ* incubations correspond with those measured at various depth ranges at this GoM marine seep (Table 2) and were not limiting (in bulk geochemistry; Table 5) over the course of the incubation. Given that these experiments were conducted at conditions that might favor anaerobic alkane oxidation, e.g., an abundance of one alkane in the gas phase, and a media replete with sulfate, these data provide insight into the relationship between alkane oxidation and sulfate reduction, and represent “potential” rates of *n*-alkane consumption linked to sulfate reduction.

With the aforementioned points in mind, we address the linkage between alkane consumption and sulfate reduction from a few perspectives. First, based on the stoichiometry of each alkane oxidation-sulfate reduction pathway (Table 6) we estimate the contribution of each oxidation pathway to SRR. Second, we use the change in the isotopic signatures of the alkanes and the DIC pool to estimate carbon exchange between the alkane and DIC pools, and compare this carbon mobility with the alkane-oxidation rates (Table S2). Furthermore, we examine the community composition within the enrichments in an effort to elucidate community members potentially responsible for alkane oxidation and sulfate reduction (Figures 3–5).

From the stoichiometry of the reaction pathways (Table 6), which assume the alkanes are completely oxidized to HCO^−^_3_, and that no alkane-derived carbon is assimilated, the linkage between alkane oxidation and sulfate reduction can be estimated. The methane incubation showed a potential net consumption rate of 263 nmol C mL^−1^ day^−1^ (Table 4). This coincides with a potential SRR of 297 nmol S mL^−1^ day^−1^ resulting in a carbon to sulfate ratio of (0.9), consistent with the stoichiometric prediction of C:S of 1. These estimates assume that all the sulfate reduction observed in the incubations is a result of methane oxidation, however, the N_2_ control treatments indicate a potential intrinsic SRR of 94 nmol mL^−1^ day^−1^. If we assume changes in the community activities between the methane addition and control treatments are negligible, aside from the oxidation of the methane, then we can use the SRR of the control treatment as a background SRR. Correcting for the intrinsic sulfate reduction results in an apparent SRR of 203 nmol mL^−1^ day^−1^ and an excess methane consumption (a C:S of 1.3, Table 4).

One way in which more carbon may be consumed than predicted from stoichiometry is if the alkane is not completely oxidized to DIC. The shifts in the isotopic composition of the DIC and alkane pools can be used to constrain how much carbon has moved from the alkane pool into the DIC pool and thus establish if another carbon sink may be important. The moles of carbon from the alkane pool needed to shift the DIC pool from its initial to final composition can be described by:

(2) $$\begin{array}{l} \delta^{13}{\text{C-DIC}}_{Tf}*[{\text{DIC}}_{T0}+\text{Alk}]*V=\delta^{13}{\text{C-DIC}}_{T0}\\ \;*\;[{\text{DIC}}_{T0}]*V+\delta^{13}{\text{C-Alk}}_{T0}*[\text{Alk}]*V \end{array}$$

where δ^13^C-DIC and δ^13^C-Alk represents the isotopic signature of the pool, [DIC_*T*0_] is the concentration of the initial DIC pool (mol C L^−1^), [Alk] is the alkane carbon oxidized (mol C L^−1^), and *V* is the incubation volume (100 mL). Initial [DIC_*T*0_] and δ^13^C-DIC_*T*0_ were calculated as a 1:1 (v:v) mixture of measured values from the media and average values from the pore water (Table 2) which comprised the slurry incubations. Using these values the amount of carbon transferred from the initial alkane pool can be estimated. For the methane incubations, this calculation suggests that 2.2 mmols C are transferred from the methane to the DIC pool over the course of the incubation, indicating a rate of methane consumption of 275 nmol mL^−1^ day^−1^. This is reasonably consistent with the rate estimates derived from the change in methane concentration over time.

When ethane oxidation is coupled to sulfate reduction, it results in a carbon to sulfate ratio of 8:7 (or 0.57, Table 6). The reported oxidation rate of 168 nmol ethane mL^−1^ day^−1^ (or 336 nmol C mL^−1^ day^−1^) would lead to the consumption of 294 nmol SO^2−^_4_ mL^−1^ day^−1^. This oxidation rate accounts for the majority (89%) of the total estimated sulfate reduction (330 nmol SO^2−^_4_ mL^−1^ day^−1^) during the incubation. When SRR are corrected for the potential intrinsic rates, it results in a corrected rate of 236 nmol SO^2−^_4_ mL^−1^ day^−1^. The observed ratio of carbon oxidized to sulfate reduced is 0.7, about 25% more carbon than expected (Table 4). Unfortunately, the analytical resolution of the δ^13^C-ethane precludes estimation of carbon movement among pools (Table S1).

In the case of propane, the estimated SRR increased 2.6-fold over the N_2_ control, with an observed oxidation rate of 354 nmol propane mL^−1^ day^−1^ (or 1062 nmol C mL^−1^ day^−1^). Given the stoichiometric relationship of 6:5 (Table 6), this consumption corresponds to 885 nmol SO^2−^_4_ mL^−1^ day^−1^, a rate 3.6-fold higher than the measured potential SRR (246 nmol S mL^−1^ day^−1^) (Table 4). However, examining the change in the DIC pool, we calculate that 3.9 mmols C (1.3 mmols of propane) moved from the propane pool to the DIC pool (Table S1). This is equivalent to an oxidation rate of 487 nmol C mL^−1^ day^−1^, accounting for 46% of the total loss of propane. Thus, there must be another sink for propane (discussed below), implicating the presence of another oxidant, another source of light carbon to the DIC, or both.

Similar to the propane treatment, the butane addition also resulted in higher SRR (2.3-fold) compared to the control treatment. Over the course of the experiment, butane was consumed at a rate of 125 nmol butane mL^−1^ day^−1^ (or 500 nmol C mL^−1^ day^−1^) (Table 4). The corresponding total (220 nmol S mL^−1^ day^−1^) and corrected SRR (125 nmol S mL^−1^ day^−1^) can only account for about one-third of the butane consumption (Table 4). However, unlike propane, the majority (85%) of the butane carbon can account for the change in the DIC pool (Table S1). Again it is possible that another sink may exist but identifying such a sink is beyond the scope of these data.

The data above underscore that the rate of oxidation of ethane, propane, and butane cannot be explained solely by the estimated rates of sulfate reduction. Similar observations have been reported by Quistad et al., who noted that the propane loss they observed might be accounted for by abiotic processes such as leakage and dissolution, partial degradation to alcohols or acids, and/or inaccuracy in measurements (Quistad and Valentine, 2011). In our study, however, the observed higher rate of *n*-alkane oxidation may be best explained by (1) utilization of oxidants other than sulfate (e.g., NO^−^_3_, which is present at site GB425 though not measurable in our reactors; Table 2), (2) errors in the estimation of either the oxidation or reduction rates or isotopic assays due to systemic errors, (3) changes in the microbial community or activity of the community over the course of the incubation that were not observable with the sampling design, or (4) the precipitation of (authigenic) carbonate in the batch reactors as has been noted to occur in GoM sediments (Sassen et al., 2004). We address each of these possibilities in detail below.

Nitrate and/or nitrite represent potential alternative oxidants for alkane oxidation, and are present at ≤40 μ M in the upper layers of Garden Banks sediments. Their concentrations were, however, below our detection limits of 0.5 μ M in the sediment slurries at both *T*_0_ and *T_f_*. Even if one assumes that 40 μ M NO^−^_3_ was present in sediments, it could only produce 50 μ M DIC (5 μmol C) through complete NO^−^_3_ reduction to N_2_. Thus, nitrate coupled alkane oxidation cannot solely explain the observed discrepancies.

To determine if alkanes were systemically lost via leakage and/or other bottle effects, sediment-free reactors were incubated in parallel, and alkane concentrations were monitored over the course of the incubations. These reactors exhibited <5% loss over the course of the incubations, which is markedly lower than our least active biological treatment (CH_4_). SRR is known to be sensitive to the incubation time, although sediments—including those from the GoM—are typically incubated for 24 h as was used here (Fossing and Jorgensen, 1989). Moreover, the rates shown here are comparable to previously published rates (Arvidson et al., 2004; Joye et al., 2004). In any case, shorter incubation times might result in higher SRR, and might account for some of the observed excess alkane consumption. Leakage of gas from sample vials (different than the incubation vials) could also affect the isotopic signatures of the alkane pools and could be a source of error resulting in lower precision measurements (though nothing in our data is consistent with this hypothesis).

Shifts in the microbial community could lead to enrichment of acetoclastic methanogens that can use acetate (a possible product of partial propane or butane degradation) to produce methane. Methane has been reported as a potential carbon sink during degradation of higher molecular weight hydrocarbons (Gray et al., 2010). However, in the present study we do not observe any changes in the concentration of the methane produced compared to the N_2_ controls (Table S2). We also do not observe an increase in the total methanogen population in the C_2_–C_4_ amended sediments by qPCR with primers specific for the methanogenic *mcrA* gene. Thus, methane appears unlikely to be a carbon sink in our experiments.

Finally, precipitation of carbonates may be a sink of carbon within the sediments. The inorganic precipitation of carbonate, known as authigenic carbonate, can occur at the sediment-water interface or within the sediment pore water. Authigenic carbonates are often formed in sediments where increasing alkalinity, typically from sulfate or metal reduction, increases the carbonate saturation state past a saturation threshold causing precipitation of minerals (calcite or aragonite). Sulfate-reduction increases the alkalinity of pore waters by removing hydrogen ion from the local environment in the form of H_2_S and generating bicarbonate concentrations by oxidizing organic carbon. Authigenic carbonates are found throughout the GoM (Roberts and Aharon, 1994; Sassen et al., 2004). Generally, environments such as the GoM with substantial amounts of organic carbon but that hinder aerobic respiration and support alkalinity-increasing processes such as sulfate reduction have the potential to harbor large carbon sinks in the form of authigenic carbonate (Higgins et al., 2009). While inorganic precipitation of carbonates is possible in our incubations, data for the total sedimentary inorganic carbon content is unavailable.

Microbial community analyses via pyrotag sequencing implicate that certain members of the class Deltaproteobacteria are enriched during the batch incubations. Further phylogenetic analyses indicate that the enriched bacteria are closely related to previously enriched/isolated C_3_–C_4_ degrading SRP (Kniemeyer et al., 2007; Jaekel et al., 2013), as well as uncultured marine SRP observed in GoM sediments (Orcutt et al., 2010; Kleindienst et al., 2012). The Deltaproteobacterial sequences most enriched in the ethane incubations were closely related to isolate BuS5, and the enrichment culture Butane12-GMe, both of which belong to the DSS cluster (Kniemeyer et al., 2007). Intriguingly, previous studies suggest that BuS5 (degrades propane and *n-butane*) and enrichment Butane12-GMe (degrades *n*-butane) do not degrade ethane (Kniemeyer et al., 2007; Jaekel et al., 2013). Other uncultured DSS cluster members were also identified in the ethane degrading incubations (Kleindienst et al., 2012). Thus, it is possible that SRP closely related to the C_3_–C_4_ degrading DSS cluster might be associated with ethane degradation in these incubations, though this hypothesis remains to be tested.

While pyrotag sequencing using 16S rRNA gene data show the phylogenetic structure of the microbial community (and is not quantitative), qPCR analysis helps us to quantitatively assess the functional potential of the microbes in the reactors. Many of the Deltaproteobaceria reduce sulfate using the dissimilatory sulfite reductase or adenosine 5′-phosphosulfate reductase enzymes (encoded by *dsrA* and *aprA* genes, respectively). The qPCR results (both *dsrA* and *aprA* gene abundance) demonstrate that the total number of SRP decrease compared to the *T*_0_ sample and the N_2_ control. However, C_2_–C_4_ consumption correlates stoichiometrically with SR, and SR rates were higher in the presence of the alkane gases compared to the N_2_ control. Collectively, these results indicate that there maybe an enrichment of a specific subset of the SRP community responsible for the consumption of C_2_–C_4_. While lower in abundance than the total SRP in the *T*_0_ sample or N_2_ control, this subset of the community likely exhibits higher specific SR activity.

The data presented here provide insight into alkane oxidation rates in Garden Banks sediments. Like previous studies, these data confirm that the addition of alkanes stimulates sulfate reduction (Figure 2, Table 4). Notably, the rates of C_2_–C_4_ consumption are comparable to CH_4_ consumption, though their stoichiometric impacts on the sulfate pool vary. For example, assuming complete oxidation, it is likely that 1, 1.75, 2.5, and 2.8 moles of sulfate are reduced per mole alkane for C_1_–C_4_, respectively. Consequently, the relative effect of C_2_–C_4_ oxidation on the sulfate pool is much greater than for methane given the observed similarity in the oxidation rates. Accordingly, alkane oxidation may represent a substantial sink for sulfate in sediments where alkanes are elevated, such as the GoM where they can constitute more than 10% of the total gas pool (Milkov, 2005). This is most likely relevant deeper in the sediments, where alkane concentrations are highest and where sulfate concentrations are lowest. In such scenarios, it is not implausible that C_2_–C_4_ oxidation might limit the availability of sulfate for methane oxidation, though this speculation requires further study. The data further constrain carbon exchange between the alkane and DIC pool, and this phenomenon should be considered when interpreting DIC isotope ratios of alkane-replete sediments.

Ethane is the next most abundant short-chain non-methane alkane at our study site (Table 3). However, previous studies have reported ethane-driven sulfate reduction at very slow rates by microbial enrichments obtained from a similar location (Kniemeyer et al., 2007). Anaerobic ethane oxidation likely involves a novel mechanism because it requires the activation of a primary carbon, in contrast to butane where secondary carbons are available (Kniemeyer et al., 2007, and references therein). Notably, we observed substantial ethane consumption over the course of these incubations [approximately two orders of magnitude higher than those reported by Kniemeyer et al. (2007), and comparable to the oxidation rates of methane, propane or butane]. The presence of ethane also stimulated sulfate reduction, which implies a relationship between these processes. Our data show anaerobic ethane utilization begins without delay, similar to other alkanes, suggesting that microorganisms are poised for ethane oxidation.

Bulk geochemical and isotopic surveys of alkanes along seeps have been used to imply microbial consumption of short-chain alkanes (Sassen et al., 2004; Orcutt et al., 2005). Our data confirm that short-chain alkanes are oxidized to DIC, likely coupled to sulfate reduction. However, both our alkane consumption rates and the isotopic shifts observed in the DIC pools suggest other sinks may exist. At thermogenic hydrocarbon seeps, these processes may have an important impact on the local carbon and sulfur cycles. The strategies used herein—namely the combination of molecular, geochemical, and isotopic assessments—was leveraged to establish the relationships between anaerobic alkane oxidation and SRRs, carbon flux, microbial activity, and microbial community composition and phylogeny. Future experiments should consider these and previous data to gain further insight into the signatures and mechanisms of these biogeochemical processes as well as organisms involved in anaerobic short-chain alkane oxidation.

## Author contributions

Arpita Bose, Melissa M. Adams, and Peter R. Girguis designed the research. Melissa M. Adams and Peter R. Girguis directed the *in situ* collections and Samantha B. Joye performed the *in situ* measurements. Arpita Bose and Melissa M. Adams conducted the batch reactor incubations. Arpita Bose and Daniel R. Rogers performed the *ex situ* geochemical analyses. Arpita Bose and Daniel R. Rogers determined the rate of alkane consumption, rate of sulfate reduction, and the molecular analyses. Melissa M. Adams performed the phylogenetic analyses. Arpita Bose and Daniel R. Rogers wrote the manuscript with input from Peter R. Girguis, Melissa M. Adams, and Samantha B. Joye.

### Conflict of interest statement

The authors declare that the research was conducted in the absence of any commercial or financial relationships that could be construed as a potential conflict of interest.
