# Supplementary material for: Geomicrobiological linkages between short-chain alkane consumption and sulfate reduction rates in seep sediments
Source: Front Microbiol. 2013 Dec 12;4:386. doi: 10.3389/fmicb.2013.00386 (PMC3860272; doi:10.3389/fmicb.2013.00386)
Supplement: Supplementary file 1 [file DataSheet1.ZIP › 68583_Girguis_Suppl_Table_1.DOCX]

**Table S1. The δ^13^C values for dissolved inorganic carbon (DIC) at the final time-point (T_f_) and the added gases at the initial (T_0_) and final time-point (T_f_).**

| **Sample** | **DIC δ^13^C at T_f_** | **δ^13^C of added gas at T_0_** | **δ^13^C of added gas at T_f_** | **Observed C-addition from alkane to DIC pool (mmols C)** | **Predicted DIC production from alkane oxidation (mmols C)*** | **% alkane C recovered in DIC** |
| --- | --- | --- | --- | --- | --- | --- |
| **Methane** | -26.3±1.5 | -39.1±0.1 | -38.2±0.6 | 2.2 | 1.6 | 137 |
| **Ethane** | -27.4±1.1 | -24.6±3.3 | -27.5±0.8 | n.a. | 2.7 | n.a. |
| **Propane** | -28.1±2.6 | -36.0±1.3 | -31.6±1.1 | 3.9 | 8.5 | 46 |
| **Butane** | -31.2±0.6 | -42.1±0.8 | -37.7±1.9 | 3.4 | 4 | 85 |

n.a. – not applicable due to poor precision in the δ^13^C-ethane measurements; DIC – dissolved inorganic carbon.

* Assuming complete oxidation of the short-chain alkane.
