# Supplementary material for: Geomicrobiological linkages between short-chain alkane consumption and sulfate reduction rates in seep sediments
Source: Front Microbiol. 2013 Dec 12;4:386. doi: 10.3389/fmicb.2013.00386 (PMC3860272; doi:10.3389/fmicb.2013.00386)
Supplement: Supplementary file 1 [file DataSheet1.ZIP › 68583_Girguis_Suppl_Table_2.DOCX]

**Table S2. Concentration of methane produced in the Nitrogen, ethane, propane and butane degrading reactors. Data shown are for n=6 i.e., 3 measurements for duplicate reactors. It should be noted that these estimates are calculated based on IRMS measurements, though similar data from obtained via GC-FID show a similar production.**

| **Reactor** | **Methane produced (mM)** |
| --- | --- |
| **Nitrogen** | 2.56±1.92 |
| **Ethane** | 1.42±0.32 |
| **Propane** | 2.21±1.93 |
| **Butane** | 0.85±0.41 |
