# Supplementary figures and images for: Geomicrobiological linkages between short-chain alkane consumption and sulfate reduction rates in seep sediments
Source: Front Microbiol. 2013 Dec 12;4:386. doi: 10.3389/fmicb.2013.00386 (PMC3860272; doi:10.3389/fmicb.2013.00386)

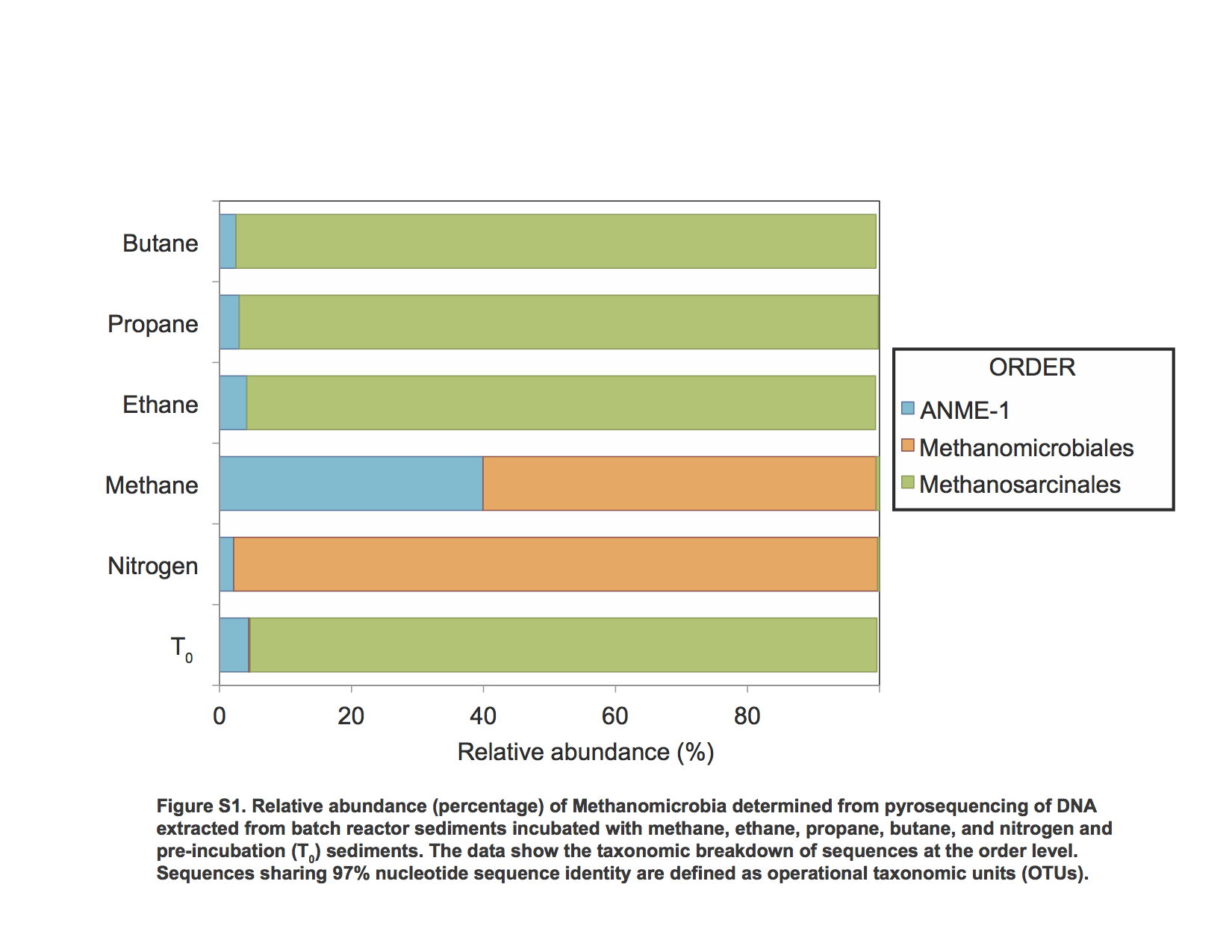

Supplement: Supplementary file 1 [file DataSheet1.ZIP › 68583_Girguis_Suppl_Figure_1.TIFF]
